# Microbial Dynamics in Endodontic Pathology—From Bacterial Infection to Therapeutic Interventions—A Narrative Review

**DOI:** 10.3390/pathogens14010012

**Published:** 2024-12-29

**Authors:** Klara Wieczorkiewicz, Anna Jarząbek, Estera Bakinowska, Kajetan Kiełbowski, Andrzej Pawlik

**Affiliations:** 1 Laboratory of Paediatric Dentistry, Pomeranian Medical University in Szczecin, Powstancow Wlkp 72, 70-111 Szczecin, Poland; klaraw@op.pl (K.W.); anna.jarzabek@pum.edu.pl (A.J.); 2 Department of Physiology, Pomeranian Medical University, 70-111 Szczecin, Poland; esterabakinowska@gmail.com (E.B.); kajetan.kielbowski@onet.pl (K.K.)

**Keywords:** endodontic infection, endodontic treatment, bacteria, enterococcus, fusobacterium, porphyromonas

## Abstract

Endodontic infection is a penetration of microorganisms into the dental pulp. Bacteria are the most common entities that induce an infection. This state is associated with significant pain and discomfort. Therapeutic intervention involves removal of infected pulp from the tooth and roots, which eliminates viable tissue, thus creating a tooth less resistant to mechanical pressure. Studies suggest that there are several types of bacteria most commonly associated with endodontic infections. Furthermore, it is considered that different types of pathogens could play a major role in primary and secondary endodontic infections. The aim of this review is to summarize major bacteria involved in the process of endodontic infection. Furthermore, we discuss the bacterial properties that allow them to penetrate dental pulp and hypothesize about possible future treatment strategies.

## 1. Introduction

Endodontics is a part of dentistry that focuses on physiology, pathology, diagnostics, prevention, and treatment of diseases: endodontia (also called pulp-dentin complex) and apical tissues of the tooth (AP). The tooth pulp and periapical tissues are anatomically, topographically, and functionally related. The causes of pulp diseases are most often caries and trauma, and existing pulp diseases can lead to pathological changes in AP tissues. Despite treating the involved tissues, therapeutic procedures used in pulp diseases prevent disorders in AP. Untreated pathological changes in AP tissues may lead to periapical bone resorption [1]. The treatment of inflamed pulp is complicated by the structure of the pulp and the anatomy of the tooth, particularly the complex canal system in the root(s). This makes it impossible to restore the pulp to its normal physiological state. To preserve a tooth affected by pulp or periapical tissue diseases, endodontic treatment is needed. This involves the preparation, disinfection, and filling of the root canals. It facilitates faster healing, halts the infection, and keeps it under control. However, it is important to note that after such treatment, the tooth does not contain vital pulp, which reduces its resistance to stress from speech, chewing, or contact with opposing teeth. Additionally, its defensive capacity decreases, which in critical situations can lead to the extraction of the tooth [2].

The global prevalence of teeth treated with endodontic therapy is 8.2%. Meanwhile, the percentage of people who have at least one tooth treated with endodontic therapy is 55.7% worldwide and 59.6% in Europe. It was also shown that the trend in the prevalence of such teeth has decreased over the years. In the 20th century, it was 10.2%, while in the 21st century it has dropped to 7.5% [3]. The frequent performance of endodontic treatment is supported by its high success rate. In 2022, Fransson et al. stated that only 2% of endodontically treated teeth are lost within a year [4]. To effectively eliminate and prevent infections in the canal system, pulp diseases must be appropriately classified. Pain and tooth sensitivity are features of symptomatic pulpitis, whereas apical periodontitis can manifest with both pain and swelling. Its chronic form may be asymptomatic and its presence is indicated by apical radiolucencies. In addition, before starting treatment, it is necessary to assess whether the tooth has a vital or necrotic pulp.

The microbial aspect is considered the primary cause of the aforementioned conditions. Therefore, during dental treatment, it is crucial to maintain proper aseptic techniques. Endodontic procedures are highly complex, requiring good lighting, using magnification (loupes, microscopes), and increasingly, due to the search for new methods, the use of lasers [1]. The most common cause of pulp disease is bacterial infection. Bacteria invade and penetrate the endodontic space through various routes, including carious lesions or trauma. The bacterial species that invade this environment are diverse, including facultative aerobes and anaerobes, as well as resilient and difficult-to-eradicate species capable of surviving in harsh conditions, such as *Enterococcus faecalis*, which contribute to recurrent periapical periodontitis. Dioguardi et al. (2019) demonstrated that *Enterococcus faecalis*, *Actinomycetes*, and *Propionibacterium propionicum* are the species most commonly involved in persistent root and extraradicular infections [5]. In addition to the previously mentioned procedures, medications are also used to eliminate bacteria. Their effectiveness has only been documented when combined with a tight seal in both coronal and apical fillings. As an adjunctive treatment, systemic antibiotics are recommended in patients with acute apical abscess with systemic involvement or who are medically compromised. Furthermore, patients with progressive infections are also recommended to receive antibiotics [6]. The challenging nature of treating the endodontic space and its anatomy emphasizes the aim of this review, which is to systematize the latest knowledge on the microbiological causes of endodontic infections and methods for managing them to reduce, eradicate, and prevent their occurrence by creating an appropriate environment [5].

## 2. Progression of Oral Diseases Leading to Endodontic Treatment

There are many causes of endodontic infections. By far the most common oral disease that leads to pulp infection and, consequently, to endodontic treatment is tooth decay (deep caries). The development of carious lesions is associated with biofilm. This happens through the metabolism of sugars provided by diet into acids by dental plaque bacteria, which causes the demineralization of the tooth’s hard tissues [7]. If there is a disturbance in the structure of hard dental tissues, untreated caries lead to infection of the pulp—the colonization of the root canal system and periapical tissues by bacteria (Gram-positive bacteria, including *Streptococci, Lactobacilli*, and *Actinomyces*) [8].

Endodontic infection can also occur as a result of trauma to the tooth [9]. This process differs from the pathway seen in caries, which usually start in the coronal region and end in the periradicular area. Trauma can occur at any level of the tooth tissues and surrounding structures. In the post-traumatic state, these tissues are less resistant to bacterial attack because the blood supply to the pulp and the periapical nervous system becomes disrupted. Additionally, some of the dental injures (tooth luxations and fractures crown and/or root) create direct pathways for bacteria from the external environment and oral cavity to invade the pulp. As time passes, bacterial invasion intensifies (initially, planktonic bacterial cells are easily eliminated by a healthy, intact host immune system, but over time, immune response capacity decreases, and areas lacking pulp vascularization due to trauma become prone to biofilm formation) [10]. Endodontic treatment is also necessary in “endo-perio” syndromes, in which there is simultaneous infection of the pulp and periodontal disease [11]. Inflammation located in the pulp can be transferred to the periodontium. On the other hand, periodontal inflammation and gingival recessions can also cause inflammation of the pulp and its necrosis [12]. Another consequence of untreated periodontal disease can be inflammatory changes in the periapical tissues [13]. Xavier-Fructuós Ruiz et al. (2017) demonstrated that in patients with periodontal disease, the risk of developing AP in teeth that have already undergone endodontic treatment is 5.19 times higher than in individuals with healthy periodontal tissue [14]. This is due to the similarity of bacterial flora, which is found in the endodontic system and pathological pockets—the dominant microorganisms are *Streptococcus, Peptostreptococcus, Eubacterium, Bacteroides*, and *Fusobacterium*. Endo-perio syndromes can occur in several forms of endodontic origin, of periodontal origin, or a combination of both [12]. The infection of this niche is still not well understood, and it is associated with the high level of difficulty in treating such infections and lesions. *T. forsythia*, *P. gingivalis*, and *A. actinomycetemcomitans*, found in diseased periodontal tissue on the external surface of the root, have several pathways to infect the pulp: through the apical foramen, lateral or accessory canals, or even the controversial dentinal tubules, which should be protected by healthy cementum (in its absence, they become exposed) [11].

## 3. Microbial Homeostasis and the Role of Bacteria in Endodontic Disorders

The oral microbiome consists of hundreds of microorganisms—bacteria, protozoa, archaea, fungi, and viruses. Among bacterial species, a group of small bacteria known as candidate phyla radiation bacteria are also frequently identified [15,16]. The oral cavity is not a uniform habitat; it contains diverse niches, including the surfaces of the buccal mucosa, lips, palate, tongue, and teeth. Moreover, the oral microbiome changes throughout an individual’s life [17]. Saliva has an important impact on the composition of oral microbiome, as it contains substances serving as nutrients and antimicrobial molecules that together shape the presence of particular species [18].

Provided proteins, carbohydrates, and nucleic acids sustain the microorganisms in the oral cavity, allowing them to occupy any space, with a preference for moist surfaces [19]. Changes in the key environmental parameters related to diet and hygiene can disrupt the natural balance of the microflora and promote cariogenic microorganisms, leading to caries, pulp diseases, gum diseases, periodontal diseases, and periapical inflammations, as the colonization of the oral microbiome mainly occurs on the surfaces of the teeth [17]. Few studies demonstrated the impact of dietary modifications on oral microbiome composition [20,21]. Oral diseases are also caused by viruses, and such infections can manifest as ulcers. In this niche, herpes virus, human papillomavirus, Coxsackie, and mumps, measles, and rubella have been detected. In the case of poor oral hygiene, protozoa are also identified in the oral cavity, the dominant ones being *Entamoeba gingivalis* and *Trichomonas tenax*. *Candida* species are the main fungi in the oral cavity; other species involve *Saccharomycetales*, *Fusarium*, *Cryptococcus*, *Cladosporium*, *Aureobasidium*, and *Aspergillus*. Oral candidiasis is a frequent opportunistic fungal infection [22]. The smallest portion of the described microbiome of oral cavity consists of archaea, which are also the least understood [17].

A healthy oral cavity is defined as maintaining a balance between microorganisms and their stable populations. Recently, it was demonstrated that *Firmicutes*, *Proteobacteria*, *Bacteroidetes*, *Actinobacteria*, and *Fusobacteria* are the most abundant phyla. The composition of the microbiome is similar across different regions in the oral cavity, with some differences. For example, *Bacteroides* and *Fusobacteria* were more abundant in tooth surface while the presence of *Firmicutes* was associated with swab samples [23]. The most commonly found species include *Streptococcus*, *Leptotrichia*, *Eikenella*, *Granulicatella*, *Actinomyces*, *Fusobacterium*, *Corynebacterium*, *Rothia*, *Porphyromonas*, *Prevotella*, *Haemophilus*, *Treponema*, *Neisseria*, *Capnocytophaga*, *Lactobacillus*, *Veillonella*, *Peptostreptococcus*, *Gemella*, *Staphylococcus*, *Eubacteria*, and *Propionibacterium*. The emergence of disorders is defined by changes in the entire community rather than just one microorganism [17]. Infection of the endodontium occurs when it is penetrated by microorganisms. The most common cause of diseases in this area is bacterial infection.

A biofilm forms through multi-stage process. Initially, pioneer bacteria adhere to a protein film that exists on all surfaces in the oral cavity, using adhesion forces [24,25]. At this stage, *Streptococci*, mainly *Streptococcus oralis*, as well as *Actinomyces naeslundii* and *Veillonella dispar*, dominate. These bacteria define a healthy oral environment; however, *Porphyromonas gingivalis* is considered pathogenic, despite sometimes being present in the primary biofilm. With excessive biomass growth (due to poor hygiene), the quantity and distribution of pathogenic microbiomes increase, leading to localized infections, as they disrupt the host’s immune system [26]. In such cases, treatment is complicated by the variable gene expression of microorganisms, which protects them from the host’s defense system. This highlights the importance of preventing biofilm growth, underscoring the need to maintain proper oral hygiene [26]. Maturation is one of the processes of biofilm formation. This leads to changes in the bacterial population; less oxygen results in a higher number of strictly anaerobic bacteria, which causes changes in the biofilm metabolism towards anaerobic fermentation [27]. Biofilm maturation significantly affects the sensitivity profile to disinfecting agents [28]. The bacteria forming the biofilm are a well-organized, cooperating community, functioning in microcolonies connected by channels, creating an ideal system for communication and exchange of genetic information, which gives them resistance to the host’s immune system as well as antibacterial agents and antibiotics [27]. Analysis of endodontic biofilm is important, as it allows for the introduction of appropriate therapy. It is extremely difficult to recreate endodontic biofilm in vitro, hence the high level of difficulty in its recognition and analysis. Unfortunately, the microbiome of this environment has not yet been thoroughly studied. Initially, it was believed to consist mainly of one species, *E. faecalis*. Advanced sampling techniques have shown that root canals are ideal habitats for many coexisting microorganisms. It is estimated that root canals typically yield between 103 to 108 bacteria, belonging to 20 different species. Infected canals may contain both aerobic and anaerobic bacteria, with strict anaerobes constituting 70–90% of the total bacterial flora in the canals due to the presence of necrotic tissue and lack of blood circulation, while any available oxygen is consumed by the resident microbiota. The data presented by Gomes et al. show that 70% of the bacterial isolates were strict anaerobes or were microphilic [29]. Studies have also shown that the proportion of obligate anaerobic microorganisms colonizing the root canal increases when the infection persists for a sufficiently long time [30].

Endodontic treatment is required for both primary (PEI) and secondary/persistent (SPEI) endodontic infection. Apical periodontitis (AP) represents an inflammatory condition of peri-radicular tissues due to invasion and colonization of bacteria in the root canals. Primary apical periodontitis (PAP) is associated with untreated necrotic root canals and can be efficiently treated with endodontic treatment to remove bacteria. Persistent/secondary apical periodontitis (SAP) is a perpetual periapical lesion due to unsuccessfully treated root canals after an initial apparent healing of the tooth. Despite similar methods being used during the procedures, these two cases show differences in the microbiota as evidenced by the literature [31]. Gomes et al. demonstrated significant differences in the microbiological composition of primary and secondary infected root canals. The microflora of the root canal in untreated teeth with AP was mixed, consisting of Gram-negative and Gram-positive microorganisms, primarily anaerobic, and typically contained more than three species per canal. In contrast, in canals with failed endodontic treatment, facultative anaerobic and Gram-positive bacteria predominated; these canals generally contained 1–2 species. The increased prevalence of Gram-positive bacteria may be due to enhanced resistance to instruments and antiseptic agents [32]. Hong et al. [33] and Tzanekis et al. [34] demonstrated similar bacterial flora in both types of infections (PEI and SPEI), with *Bacteroidetes* being the dominant species. Vengerfeldt et al. also showed that *Firmicutes* and *Bacteroidetes* were the most numerous in both PEI and SPEI [35]. In the recent study by Park et al., no significant differences were observed in the diversity and abundance of organisms between the two types of infections. In both groups, the dominant phyla were *Proteobacteria*, *Firmicutes*, *Fusobacteria*, *Bacteroidetes*, and *Actinobacteria* [31]. Other studies indicate that the primary types of bacteria in PEI include *Prevotella*, *Fusobacterium*, *Porphyromonas*, *Parvimonas*, and *Streptococcus*, while the most numerous genera include *Firmicutes*, *Bacteroidetes*, *Proteobacteria*, *Actinobacteria*, and *Fusobacteria*. Abusrewil et al. demonstrated that the endodontic microbiome exhibits variability depending on the sampling site within the root canals. They found that 99% of its composition is made up of *C. albicans* and *S. gordonii*, with low counts of *F. nucleatum* and *P. gingivalis*. In summary, researchers proved that by influencing *C. albicans*, one can gain control over the rest of the endodontic microbiome [29]. Park et al. also observed a relationship between the microbial composition of the canals and the gingival sulcus in SPEI. Specifically, specific organisms were present in gingival sulcus and canals, including species such as *Oribacterium*, *S. salivarius*, *L. parvula*, and *P. denticola*, which may serve as diagnostic biomarkers for the non-invasive detection of SPEI [31].

These differences can be explained by factors such as the race and ethnicity of the patient, geographic location, and even the use of different DNA sequencing methods. It is also important not to overlook *E. faecalis*, which is mentioned regarding its predominant occurrence in the secondary infection by Tennert et al. [36]. This bacterium is capable of surviving in harsh conditions, has high virulence factors, and is aggressive towards dentinal tubules, which translates to its resistance to the host’s response and its presence in chronic AP inflammation [31]. It can survive in an environment with scarce nutrients and minimal coexistence with other bacteria. Its virulence factor in previously endodontically treated teeth may be related to the ability of *E. faecalis* to invade dentinal tubules and adhere to collagen in the presence of human serum [32]. The presence of *E. faecalis* in SPEI was confirmed by Vengerfeldt et al. [35]. Foschi et al. demonstrated that *E. faecalis* was typical for secondary, asymptomatic chronic AP, and its presence was correlated with treatment failures, which was also confirmed by Gomes [30]. Recently, using a modern high-throughput sequencing technique, Alquria et al. [37] demonstrated a diversity of bacteriome in patients with PEI with AP. Researchers analyzed material from 27 patients and 1 negative control, and found 9 bacterial phyla. Among them, the top 10 abundant genera were *Prevotella*, *Porphyromonas*, *Fusobacterium*, *Bacteriodaceae*, *Parvimonas*, *Fretibacterium*, *Pyramidobacter*, *Dialister*, *Olsenella*, *and Peptostreptococcus*. Interestingly, the authors used data about bacteria to identify clinical biomarkers. They observed changing profiles of bacteria in patients depending on age, sex, smoking, presence of symptoms, and lesion size. For instance, researchers linked lesions greater than 5 mm with *Prevotella*, while smaller lesions were linked with *Peptostreptococcus*. Bacteria found in root canal infections express genes associated with antibiotic resistance [38]. Combining the data about microbiome diversity and antibiotic resistance could be used in the future for therapeutic or regenerative purposes.

According to Foschi et al., the presence of more severe forms of periapical tissue diseases may be attributed to *T. denticola*, which was most frequently detected in cases of symptomatic chronic periapical inflammation. According to the researchers, it is involved in bone resorption during endodontic infections [30]. The results of the study by Arias-Moliz et al. indicate that another species, *F. nucleatum*, plays a key role in SPEI. It demonstrates an important ability to co-aggregate, which is based on the presence of several adhesins. These allow *F. nucleatum* to act as a bridge between primary colonizers (e.g., *Streptococcus* spp.) and secondary anaerobic colonizers (such as *Tannerella* spp. and *Porphyromonas* spp.) of host tissues. It induces necrosis and apoptosis of neutrophils, which promotes co-aggregation of late colonizers, such as *P. gingivalis*. Additionally, *F. nucleatum* stimulates Toll-like receptor 4 (TLR4)-mediated responses, inducing an exacerbated inflammatory state. Such impairment of the host’s immune response may allow for the growth of bacteria, while the resulting inflammatory environment may provide nutrients for the community [39].

Interestingly, *E. faecalis* inhibits the growth of other microorganisms, such as *A. oris*, *F. nucleatum*, *S. anginosus*, and *P. gingivalis*, in the biofilm of HA discs. *S. aureus* exhibits similar characteristics and is also characterized by the displacement of other species. These studies allow for further analysis of the microbiome, which is difficult to access and needs to be explored to implement appropriate treatment methods [40]. Bacteria in untreated or poorly treated root canals spread to the periapical area. By studying a two-species biofilm model in vitro—reflecting the conditions around the apex—it was found that co-infection with *C. albicans* and *E. faecalis* leads to an increased risk of periapical inflammation, bone loss, and enlargement of the lesion [41].

In the study by Gomes et al., acute pain symptoms, pain in the medical history, tenderness upon palpation, and swelling were associated with the presence of Gram-negative species, especially *Prevotella*, *Porphyromonas* spp., and *Fusobacteria*. Gram-negative bacteria contain endotoxins, which can stimulate the production of bradykinin, a strong pain mediator. This is likely responsible for painful exacerbations during endodontic treatment. They also observed a correlation between the occurrence of pain symptoms and wet canals with the presence of *P. micros*. This Gram-positive coccus is also associated with both endodontic and periodontal abscesses. In this study, purulent exudate was statistically linked to *P. gingivalis*, *P. endodontalis*, *P. micros*, *F. nucleatum*, and *S. sanguis* [32].

Persistent endodontic infections are polymicrobial. It is known that the survival of bacteria in various environmental conditions of the treated root canal and periapical tissues depends on the virulence of the community and the available nutrients. Thus, bacteria with the ability to invade cells and proteolytic activity—such as *Porphyromonas* and *Fusobacterium*—have been found in high relative abundance in chronic infections. Similarly, the high prevalence of species possessing flagella, which activate TLRs that induce an inflammatory response, is associated with symptomatic infections after treatment, cross-feeding, and the release of antimicrobial compounds, leading to synergistic and antagonistic effects. Another mechanism involves the presence of key taxa. Key species of microorganisms are strongly associated taxa that significantly impact the structure and functioning of the microbiome, regardless of their abundance in space and time. These microorganisms play a crucial role in the pathogenicity of communities, and their removal can lead to drastic changes, such as disrupting the functioning of the microbiome and altering the virulence of the consortium. In periodontal disease, *Porphyromonas gingivalis* acts as a key taxon, causing tissue destruction and the release of nutrient-rich exudates, which leads to dysbiosis and promotes further growth of pathogens [39].

## 4. Key Bacterial Species in Endodontic Infections

### 4.1. Gram-Positive Bacteria

Gram-positive bacteria are significant in the development of endodontic infections. The most frequently appearing species in endodontic infections are *Streptococcus*, *E. faecalis*, and *Actinomyces*. *Streptococcus* spp. include *Streptococcus mutans*, *Streptococcus sanguinis*, *Streptococcus anginosus*, and *Streptococcus mitisi*. Bacteria lead to endodontic infections through various mechanisms, such as formation of biofilm, collagen-binding proteins, and resistance to antimicrobial treatment [42,43]. These mechanisms allow bacteria to adhere to the surface and form conglomerates that are difficult remove mechanically and are resistant to endodontic treatment.

*Streptococcus* is bacterial genus that belongs to the *Streptococcaceae* family. The size of bacteria ranges between 0.5 and 2 µm. They are facultative anaerobes that commonly arrange in chains or in pairs [44]. *Streptococcus mutans* plays a significant role in the development of endodontic infections. Its virulence is a consequence of biofilm formation, ability to adhere to collagen, and toleration of low concentrations of oxygen, as well as the ability to produce great amounts of organic acids [45,46]. Biofilm formation is an important mechanism in the development of endodontic infections. *S. mutans* produces many compounds, allowing it to adhere to the surface of the tooth and to form dental plaque, for instance glucan-binding proteins (Gbps), collagen-binding proteins, antigen c, and glucosyltransferases (GTFs). Gbps are proteins that mediate the binding of bacteria to glucan. The literature describes four types of Gbps: GbpA, GbpB, GbpC, and GbpD [47]. GbpA is responsible for binding exopolysaccharides and proteins to build biofilm and maintain an environment conductive for its further development. The absence of GbpA results in weakened, unstable, and heterogenous structure of biofilm [48]. GbpC was described as a protein associated with cell walls, and functions as a receptor for glucan on *S. mutans* surface. It was suggested that its absence cloud result in reduction of its caries-causing properties [49].

Collagen binding-protein owes its name to the ability to bind with type I collagen, a significant element of dentin [50]. The most common collagen-binding proteins are Cbm and Cnm proteins [51,52]. It was observed that strains with cnm gene were present in approximately 12,4% of oral isolates in a Thai population [53,54]. Interestingly, the Cnm gene was found in asymptomatic endodontic infections [43].

*S. mutans* synthesizes three classes of GTFs, named GTFB, GTFC, and GTFD. Glucosyltransferase B is an enzyme that hydrolyzes sucrose present in diet into glucan. Simultaneous production of glucan by both GTFC and GTFB allows the formation of a matrix that increases the ability of bacteria to adhere to the tooth enamel and to each other. This process allows the creation of high-density biofilm [55,56,57]. This makes mechanical clearance of the bacteria more demanding. The formation of biofilm allows bacteria to survive in variable environments, including pH changes or nutrient limitation. Moreover, *S. mutans* has the ability to tolerate low pH concentrations due to presence of ATPase, which translocates protons, causing acidification of the environment and thereby creating a more competitive environment for other microorganisms. Additionally, *S. mutans* can generate bacteriocins named mutacins, which are crucial for the production of biofilm [58,59,60].

**Figure 1:**
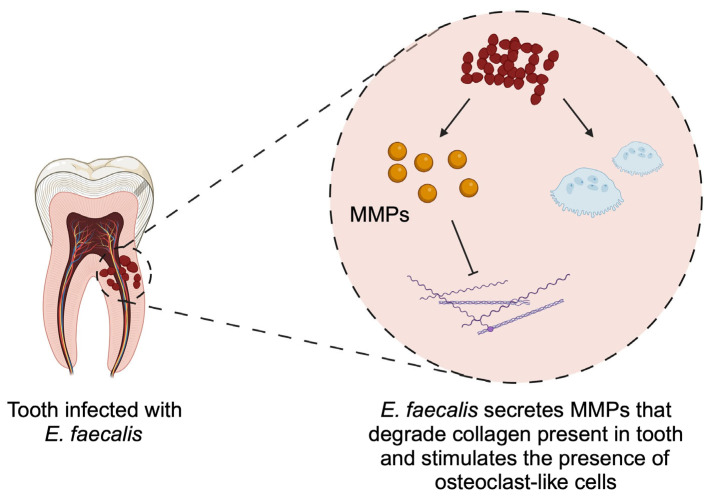
Enterococcus faecalis stimulates degradation of collagen and bone, thus leading to damage of tooth and surrounding bone. Created in BioRender. Kiełbowski, K. (2025) https://BioRender.com/a06x267.

*E. faecalis* is a facultative anaerobe that normally inhabits the gastrointestinal tract. The presence of *E. faecalis* in the oral cavity is suggested to have an exogenous route of origin [61]. It can be a nosocomial infection or related to contaminated food intake [62]. Importantly, the oral cavity can act as a reservoir of virulent *Enterococci* [62,63]. Compared to healthy patients, increased presence of *E. faecalis* is observed under pathological conditions in the oral cavity [64]. Necrotic tooth is an ideal environment for development of this bacteria [65]. *E. faecalis* prevalence in endodontic infections ranges between 24 and 77% [66]. In PEI, *E. faecalis* occurs in 67.5% of cases, while in SPEI, its prevalence occurs in around 89.6% [67]. It is simple to eradicate a small amount of *E. faecalis*, but greater amounts are difficult to eliminate. *E. faecalis* was identified during failed endodontic therapy in root canals during retreatment. It was suggested that it may be caused by *E. faecalis*’s ability to invade dental tubules and to attach to collagen in the human serum environment [68,69]. Moreover, *E. faecalis* can form biofilms, which act as protection for bacteria against immunological responses of organism and antibiotics [70,71]. It was discovered that origination of biofilm is controlled by three main mechanisms: sensing of quorum, small non-coding RNAs, and cyclic dinucleotide signaling [72]. It was reported that *E. faecalis* is resistant to sodium hypochlorite with concentrations above 5% [73]. Interestingly, it poses resilience towards increased pH levels, which enables its growth in the presence of calcium hydroxide (Ca(OH)_2_), which is commonly used in endodontics [74]. Apart from tolerance to high pH up to 11.5, *E. faecalis* grows in an environment with a wide range of temperatures, as well as an environment that is oxygen deprived and has limited access to nutrients [72]. In this context, it is interesting to note that periodontal ligament and serum from dentin serves as source of nutrition for *E. faecalis* [75,76]. *E. faecalis* presents resistance to antibacterial treatment. It is completely resistant to metronidazole, and some strains were observed to evolve resistance towards penicillins [77]. Furthermore, the bacteria releases metalloproteinases (MMPs), which are able to degrade dentinal collagen [78]. Additionally, the inhibition of MMP is associated with reduced viability of *E. faecalis* [79], thus suggesting the use of MMP inhibitors in *E. faecalis* infection. Considering the role of *E. faecalis* in suppressing osteoblasts [80] and enhancing osteoclast-like cells [81], the presence of this bacteria in root canals can significantly damage bone formation or regeneration (Figure 1).

Other bacteria present in endodontic infections include *Propionibacterium* [82] and *Actinomyces*. The latter species play a significant role in the development of persistent and recurring periapical disease related to endodontic therapy. *Actinomyces* spp. are an important part of normal oral flora. It was discovered that they are involved in the process of periodontitis and caries due to ability to form biofilm. Biofilm formation prevents bacteria from being removed by cleansing organisms such as saliva flow [83,84]. Interestingly, the presence of *Actinomyces* spp. can alter immune responses in the root canal. Specifically, stimulation of peripheral blood mononuclear cells with *Actinomyces* spp. was found to enhance the release of IL-8 [85]. Other studies demonstrated that root canal debridement reduces the levels of IL-8 [86,87], thus suggesting potential involvement of this cytokine in endodontic infection.

### 4.2. Gram-Negative Bacteria

Gram-negative anaerobic bacterium *F. nucleatum*, with its characteristic growth and survival requirements, is foundational for biofilm formation in the oral cavity [88]. It is associated with endodontic infections, causing pulp necrosis and periapical periodontitis. The higher its concentration, the more severe the disease, leading to advanced inflammation [89]. Virulence factors like dysregulation of inflammasomes in dental pulp cells play an important role in this infection [88]. The presence of this bacterium in the condition provides information about the microbiological composition and pathogenicity of the infection, which helps in selecting the appropriate treatment. For a gram-negative bacterium to survive in specific conditions, the biosynthesis of lipid, also known as the Raetz pathway, must be maintained. Gram-negative bacteria are characterized by having a layer of lipopolysaccharide (LPS) on their outer membrane, which serves as protection against the external environment and provides structural stability. It also regulates the passage of both hydrophilic and hydrophobic molecules through the bacterial membrane [89].

The activity of *F. nucleatum* increases when it is present with other anaerobes [90]. It is often found along with *E. faecalis*, whose growth it influences. Despite complete eradication during treatment, *F. nucleatum* can leave lysed cells in the biofilm or dentinal tubules, which act as chromosomal or plasmid DNA donors. In this way, other microorganisms acquire antibiotic resistance and virulence through the pheromones produced, leading to an increase in their pathogenicity. Additionally, it has been proven that the coexistence of *F. nucleatum* with other bacteria enhances and facilitates the growth of *E. faecalis*. This bacterium is observed in almost half of root canal infections with periapical rarefaction. It is adapted to survive in harsh conditions, and even if only 10% serum is left between appointments, it can survive and continue to reproduce [90]. The prevalence of *F. nucleatum* is attributed to several virulence factors, including adhesins, endotoxins (LPS), production of ammonia and butyrate, outer membrane proteins (Fap2, RadD), and the secretion of serine proteases [91]. The invasion of cells—lymphocytes, polymorphonuclear neutrophils, erythrocytes, epithelial cells, and fibroblasts—as well as adherence and consequently colonization, triggering the host’s mechanical response, is ensured by adhesins, particularly FadA (Fusobacterium adhesin A) [92]. Serine proteases can suppress the nutritional needs of other organisms while simultaneously damaging host cells and destroying IgA. LPS influences inflammatory states and bone resorption in a stimulatory manner by triggering the production of interleukin 1α (IL-1α) and tumor necrosis factor-α (TNFα) [90,91]. The limitation of gingival fibroblast formation is caused by the presence of ammonia and butyrate, while RadD and Fap2 induce apoptosis in leukocytes, also acting as adhesins [91]. Additionally, *F. nucleatum* causes the expression of matrix metalloproteinase 13 (MMP-13) and MMP-1 in host cells. Therefore, *F. nucleatum* is mostly associated with the extraradicular biofilm, which is adapted for horizontal gene transfer. It provides space and is a pathway for communication through plasmid transfer among microorganisms involved in endodontic infections. This indicates that *F. nucleatum* can penetrate the dentin, making it invasive towards the dentinal tubules and capable of adhering to their walls. A study by Ganesh et al. demonstrated that this bacterium exhibits significant invasiveness and can penetrate almost the entire thickness of dentin in the apical region. Additionally, it causes symptomatic endodontic infections due to coaggregation with other bacteria. The authors emphasize the need for appropriate canal irrigation agents and endodontic medications that can penetrate the dentinal tubules to reach the bacteria [90]. *F. nucleatum* infection induces the production of cytokines IL-1β along with synergistically acting TNF-α and IL-17, which cause an increase in the expression and production of other cytokines and defensins [92]. Consequently, immune cells accumulate, leading to bone resorption in the later stages. TNF-α, IL-17, and IL-1β facilitate the activation of osteoclasts. Additionally, *F. nucleatum* infection upregulates the expression of interferon γ (IFN-γ), which also contributes to the activation of cells that cause bone loss [3,92].

*P. gingivalis*, a gram-negative anaerobic bacterium, is one of the more commonly encountered bacteria in endodontic infections. It exhibits the ability to invade dentinal tubules [93]. The ability of microorganisms to cause virulence and the availability of nutrients in their surrounding environment are crucial for the survival of bacteria [94]. This explains the high occurrence of *Porphyromonas* in chronic endodontic infections due to their proteolytic ability and skill in invading cells. Understanding the functioning of these infections is important for establishing the mutual interactions of the microorganisms that cause them, such as cross-feeding or the release of antimicrobial compounds, which lead to coexistence in either a synergistic or antagonistic manner [39]. The virulence factors of *P. gingivalis* include LPS, hemagglutinins, fimbriae, hemolysin, outer membrane vesicles (OMV), and gingipains [95]. The latter disrupt the host’s immune response, alter cell signaling and function, and cause the degradation of fibrinogen, as well as cells and proteins. LPS increases inflammatory responses and stimulates bone resorption. OMV initiate the production of pro-inflammatory cytokines, pyroptosis in macrophages, and inflammasome signaling [91]. There are two types of fimbriae present: FimA (long, encoding the fimbrial protein FimA) and Mfa1 (short, polymerizing on the axis of the fimbria). They are filamentous, proteinaceous extensions that protrude from the surface of the cell and serve to adhere to host tissue, forming a biofilm and coaggregating with other cells and bacteria. Various genotypes of FimA were detected in *P. gingivalis* obtained from patients with endodontic infections [96]. FimA stimulates fibroblasts, macrophages, and epithelial cells of the gums to produce pro-inflammatory cytokines IL-1, TNF-alpha, and IL-6, which are responsible for bone resorption by differentiating osteoclasts [97].

**Figure 2:**
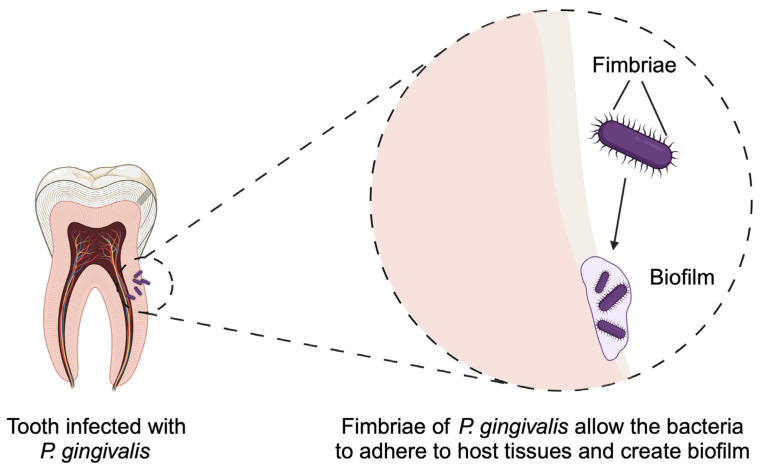
Fimbriae are an important virulence factor of *Porphyromonas gingivalis*, as they allow the bacteria to adhere to host cell and form biofilm. Created in BioRender. Kiełbowski, K. (2025) https://BioRender.com/k70t499.

The increase in the activity and diversity of osteoclasts due to the fusion of hematopoietic mononuclear progenitor cells leads to the destruction of bone tissue. *P. gingivalis*, through LPS, stimulates the formation of periosteal osteoclasts by inducing RANKL in osteoblastic cells via TLR2 activation, resulting in the loss of alveolar bone [98]. *P. gingivalis* can attack epithelial cells of the gums, the immune system, osteoblasts, and fibroblasts of the periodontal ligament. The bacterium obtains energy through the fermentation of amino acids and is a key pathogen in altering the normal composition of the microbiota into one with greater pathogenicity, thereby accelerating bone loss. *P. gingivalis* has the ability to weaken and deceive the host’s immune system [91]. We have previously mentioned that other bacteria enhance the growth of *F. nucleatum*. *P. gingivalis* could represent such microorganisms, as coculture of these pathogens was associated with significantly greater biofilm mass than that of *F. nucleatum* monoculture [99]. Due to its abilities, it is present in asymptomatic untreated teeth with chronic periapical changes (Figure 2). Therefore, further research is crucial to elucidate the antimicrobial potential of drugs used in root canals to eliminate this and other bacteria [93].

Other bacteria found in necrotic teeth involve *Treponema denticola* and *Tannerella forsythia*. Although *T. denticola* is more commonly associated with periodontal disease, in an early study by Cavrini et al. [100], the authors detected *T. denticola* in 34 out of 102 endodontic samples. In another study, Ozbek et al. [101] found the presence of *T. denticola* in the majority of cases with apical abscesses. In an in vivo experiment, infection of pulp with *T. denticola* was associated with the formation of orofacial abscesses, thus confirming the link between the bacteria and severe course of infection [102]. More recent studies evaluated potential immunoregulatory mechanisms regulated by *T. denticola*. For example, the bacteria were found to enhance the secretion of oncostatin M, a pleiotropic cytokine with increased levels in inflammatory diseases and pathological conditions associated with bacterial infection [103]. Interestingly, *T. denticola* also enhances the progression of oral squamous cell carcinoma [104], further demonstrating its various pathological properties. *T. forsythia* represents another pathogen found in necrotic pulp tissue [105]. Interestingly, the bacteria were recently associated with the occurrence of previously mentioned endo-perio syndromes [106].

## 5. Antibiotic Treatment in Endodontics

Antibiotics are considered as supportive treatment in endodontics. They are mostly used in systematic or severe infections. This is because most of the endodontic infections are limited to the tooth, and as such they can be easily treated with drainage, local surgical treatment, or tooth extraction [107,108].

However, the usage of antibiotics should be prudent due to numerous adverse effects, allergic reactions, and antibiotics resistance, which is becoming an increasing problem. In total, 8–10% of all prescribed antibiotics in primary care in developed countries are ordered by dentist [109]. Prescribing antibiotics is only empirical, as dentists do not perform antibiograms of samples form periapical region or root canal. Consequently, antibiotics should only be only prescribed when it is known that the patient will benefit from such therapy. Main indications for antibiotics use in endodontics are acute apical abscess with systemic involvement or, in immunocompromised patients, progressive infections and trauma of soft tissues that require therapy [110,111,112]. The guidelines should be strictly followed by dentists to prevent the overuse of antibiotics [113]. Endodontics infections include gram-negative and gram-positive bacteria, which can be strict anaerobes or facultative anaerobes. This indicates the need to use broad-spectrum antibiotics. Recently, Mendez-Millan et al. [114] published a systematic review and meta-analysis that investigates the use of antibiotics in patients with apical periodontitis. The authors concluded that antibiotic prescriptions are inadequate in a significant proportion of endodontic cases, thus highlighting the need to follow guidelines.

The most popular broad-spectrum antibiotics prescribed for patients are beta-lactam antibiotics like penicillin v and amoxicillin. The recommended dosage of drugs administered orally for penicillin V and amoxicillin loading dosage are 1000 mg; the maintenance doses are 500 mg every 4–6 h and 500 mg every 8 h, respectively. If the treatment with penicillin or amoxicillin fails, the next step is to start treatment with amoxicillin with clavulanic acid or penicillin with metronidazole. Clindamycin is the antibiotic of first choice for patients allergic to beta-lactams. Other antibiotics ordered for allergic patients are clarithromycin and azithromycin [6,115]. It is recommended that patients undergo a treatment lasting 3–7 days [116]. Triple antibiotic paste (TAP) is a mixture composed of ciprofloxacin, metronidazole, and minocycline in ratio of 1:1:1. Ciprofloxacin is used to fight gram-positive and gram-negative bacteria while metronidazole covers the spectrum of anaerobic bacteria; minocycline also shows activity against gram-negative and gram-positive bacteria and possesses bacteriostatic features [117,118]. However, the use of minocycline is associated with discoloration, which may be considered as drawback of the paste. Discoloration may be prevented by replacement of minocycline with cefaclor [119].

Paste can be used in vital pulp therapy, treatment of open apex teeth with necrosis, as intracanal treatment for root fracture or periapical lesions, and in regeneration and revascularization protocols. Moreover, TAP may be also used in preparation of appropriate matrix for future required treatment [120]. It is used as an antiseptic agent to remove microorganisms from radical systems. Apart from bacteria elimination, it allows the increase of anesthesia and to alleviate pain after treatment [121]. It is advised to use TAP in a 1 mg/mL concentration to prevent undesired events on stem cells of apical papilla [122,123]. The modified triple antibiotic paste (MTAP), which is a mixture of tetracycline, ciprofloxacin, and metronidazole, was discovered to be more effective in deeper levers of dentin [124]. Antibiotics prophylaxis in endodontists is not routinely required; however, in certain cases, such as imparted immunological function, risk of developing endocarditis, or for patients whose bones of jawline were exposed due to head and neck cancer with high-dose radiation, this approach is required [110,125,126].

## 6. Probiotic Approaches in Endodontic Treatment

Probiotic treatment is an innovative and promising therapy. It concentrates on the beneficial features of microorganisms in endodontic treatment such as intercanal medicament, post-treatment recovery, and as a prevention of reinfection. The use of probiotics focuses on maintaining microbial equilibrium and replacing pathogenic bacteria with beneficial ones. *Lactobacillus* and *Bifidobacterium* are species commonly used as probiotics [127]. Probiotics mechanisms of action include change of local pH, competing with other microorganisms for nutrients, production of inhibiting substances, and modulate immune system by increasing activity of immune cells. Moreover, *Lactobacillus* produce many antimicrobial substances, including hydrogen peroxide, diacetyl, organic acids, and bacteriocins [128]. Bohora et al. demonstrated that species like *Bifidobacterium* and *Lactobacillus* provided effective protection against the growth of *E. faecalis*. Moreover, the use of poloxamer can be used as probiotic carrier for intracanal endodontic agents [129]. It is worth noting that oral administration of *Lactobacillus reutei* resulted in a decrease of microorganisms in subgingival microbiota. However, this observation was not supported by clinical effects [130]. Recent clinical trial presented promising outcomes, as a three-month supplementation of *Streptococcus salivarius* M18 resulted in the decline in the accumulation of biofilm and a significant decrease in gingival bleeding. It was suggested that for maintaining such effects, patients would have to face long-term intake of probiotics [131]. Research from Mrinalini et al. reported that intercanal irrigations with *Lactobacillus* species were safer and more effective against *E. faecalis* than sodium hypochlorite [132].

## 7. Novel Strategies in Endodontic Treatment

**Figure 3:**
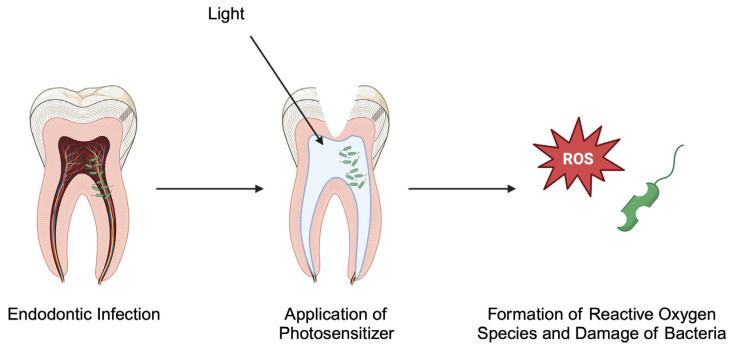
Photodynamic therapy involves the application of a photosensitizer, which reacts to light or ultraviolet by secreting reactive oxygen species that damage bacteria. Created in BioRender. Kiełbowski, K. (2024) https://BioRender.com/o88z281.

Despite the high success rate of the current strategy to treat root canal infections, the process of cleaning and disinfecting root canals can be challenging in patients with different anatomy or in those infected with bacteria that show resistance to antimicrobial compounds. Over the years, researchers have been investigating other potential methods to improve endodontic treatment. One of the novel methods is an antimicrobial photodynamic therapy (aPDT). Visible or ultraviolet lights interact with photosensitizers to create free radicals, thus damaging the bacteria (Figure 3). Recent studies demonstrated the importance of concentration of photosensitizers and combination with the adequate energy of light. In an in vitro experiment, rutin-Ga(III) complex was found to eradicate *E. faecalis* biofilm [133]. This type of therapy has been investigated clinically, which has been described in case reports and larger clinical studies. For example, Tavares et al. [134] described a patient in whom apical patency was unavailable due to a calcified and curved canal. The use of aPDT with 0.005% methylene blue solution as photosensitizer during root canal treatment was associated with uneventful post-treatment history and complete healing was noted after 12 months. Larger clinical data suggested that aPDT is a beneficial adjuvant to the conventional treatment strategy [135,136]. Recently, studies have tried to improve the efficacy of aPDT, as the therapy could be associated with enhanced production of ROS. Furthermore, hypoxic conditions present in the root canals could limit the efficacy of this oxygen-dependent method. One of the potential strategies to improve the outcomes of this treatment is combination with gas, particularly with nitric oxide (NO), which has antimicrobial properties and mild toxicity profile. Zeng et al. [137] investigated the use of CGP nanoparticles that utilize a guanidinylated poly (ethylene glycol)-poly (ε-Caprolactone) polymer loaded with photosensitizer. The aPDT triggers the release of NO. After studying the molecule in vitro, the authors analyzed its potential benefits in vivo using a rat AP model infected with *E. faecalis*. Apart from eradicating biofilms, the combination enhanced regeneration of bone defects, which demonstrates important benefits of such treatment strategy. Moreover, to counteract hypoxic conditions, a combination of aPDT with a self-supplied oxygen system was suggested [138]. Intriguingly, aPDT has recently been found to reduce the intensity of postoperative pain after endodontic intervention. The analgetic effect of the treatment was attributed to the potential of laser to reduce inflammatory responses [139].

Another promising approach to damaging residual bacteria could be the use of bacteriophages. Phages are viruses that can kill bacteria and eradicate biofilms [140]. Interestingly, Chen et al. [141] evaluated the potential efficacy of *S. mutans* mutant containing bacteriophage T4 RNA ligase 1 (T4 Rnl1). The modified bacteria demonstrated reduced capabilities to form biofilm.

## 8. Conclusions and Future Directions

To conclude, several types of bacteria were associated with the occurrence of endodontic infections. These bacteria express adhesion molecules, produce biofilm, and degrade pulp tissue, which is associated with difficulties in extracting the bacteria. Furthermore, infiltrated bacteria induce inflammatory responses and upregulate the expression of MMPs, thus damaging periapicular tissues. Over the years, studies revealed important bacterial mechanisms that are involved in the pathogenesis of endodontic infection. Further studies should analyze the behavior of less common bacteria found within root canals. Moreover, studies should explore the properties of bacteria that are strictly linked with severe course of infections, such as the formation of abscesses. Understanding the complex mechanisms induced by bacteria, together with their interactions with immune cells, could introduce targeted treatment strategies in the future.

Current treatment methods effectively eliminate bacterial infections. Nevertheless, more modern treatment methods are required in patients in whom standard techniques do not allow for a proper disinfection and debridement of root canals. Additionally, currently used methods remove vital pulp and create more fragile teeth. Future methods will potentially be able to eliminate endodontic infection and preserve viable pulp.

## Data Availability

Not applicable.
